# Metrics and methods for moving from research to innovation in energy storage

**DOI:** 10.1038/s41467-022-29257-w

**Published:** 2022-03-22

**Authors:** Sebastian Pohlmann

**Affiliations:** Skeleton Technologies OÜ, Valukoja 8, 11415 Tallinn, Estonia

**Keywords:** Energy storage, Electrochemistry, Materials for devices, Supercapacitors, Batteries

## Abstract

Research activities are crucial for the advancement of energy storage technologies. However, not all the research lead to practical innovation. Here the author, focusing on supercapacitor devices, discusses the most challenging aspects to be considered to deliver practical innovation from fundamental research.

## Teaser

Although all innovation is based on research, not all research leads to innovation. This commentary article aims to give food for thought on the importance of metrics in turning research into innovation, focusing on the evolution of supercapacitor devices as a case study.

## A general point on how to turn research into innovation

It is important to understand that all innovation begins with research, but not all research is innovative. Indeed, innovation is an idea that has a practical impact on our daily reality. That, in turn, means that an idea (and the research activities associated with it) is not entirely innovative until a practical and beneficial effect on human lives, societies or the environment is demonstrated. It also means that research does not necessarily result in immediate innovation. For example, research projects operating at low technology readiness levels usually generate scientifically accurate and methodically correct discoveries. However, in most cases, these research works cannot immediately impact the day-to-day reality, although, in the long-term, they could become relevant for future innovations that are not yet foreseeable during the initial research period.

In this commentary article, using supercapacitor technology as an example, the author aims to answer the question: which ground rules can be followed to spark innovation from research? To answer, three aspects should be considered: (i) the topic of research activities, (ii) the methods applied and, (iii) the approach to publishing.

## Research towards increasing energy density

While the selection of research topics is nearly endless, choosing a research topic that is likely to spark innovation is not a game of chance. One approach is to identify a topic that addresses an existing technology’s problem or shortcoming.

Industrial supercapacitors are energy storage devices with low energy density (ca. 10 Wh/L) and high power density (ca. 30 kW/L). They carry out millions of charge-discharge cycles and thus offer a long application lifetime. Moreover, they do not utilize lithium, cobalt, nickel or copper^1^ and are easily recyclable.

**Fig. 1:**
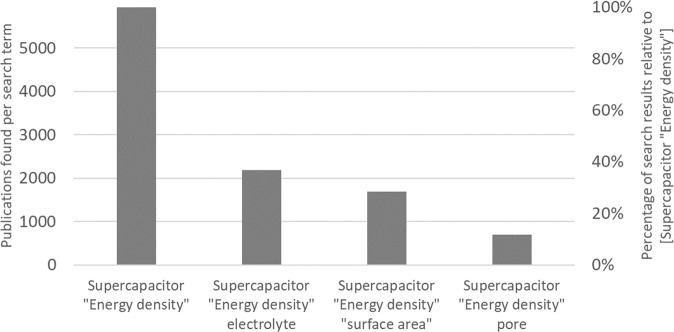
Number of publications in the field of supercapacitors focusing on different methods for energy density increase. Data collected in January 2022 using the Mendeley database and selecting the year interval 2019–2022. The searched terms were: (i) [Supercapacitor] (resulting in 13,287 total publications – value not shown in the graph), (ii) [Supercapacitor “Energy density”] (resulting in 5945 publications, i.e., 45% of the [Supercapacitor] result), (iii) [Supercapacitor “Energy density” electrolyte] (resulting in 2189 publications, i.e., 37% of the [Supercapacitor “Energy density”] result), (iv) [Supercapacitor “Energy density” “surface area”] (resulting in 1684 publications, i.e., 28% of the [Supercapacitor “Energy density”] result) and, (v) [Supercapacitor “Energy density” pore] (resulting in 699 publications, i.e., 12% of the [Supercapacitor “Energy density”] result).

Considering these aspects, it is clear that supercapacitors have multiple advantages but also an evident weakness, i.e., the low energy content. Thus, an innovative research topic that could be considered is the improvement of the energy density. This aspect is acknowledged by the scientific community considering that over 1/3 of peer-reviewed publications in the field of supercapacitors investigate the increase of the energy density, based on a rudimentary analysis of literature search results (Fig. 1). The analysis also indicates how different approaches for an energy density increase in supercapacitors receive different attention.

There are several ways to increase the energy density in supercapacitors. For example, the energy content of a capacitor is calculated by applying the following eq.:

1 $$E\left[{{\mbox{Ws}}}\right]=0.5\cdot C[{{\mbox{F}}}]\cdot {U}^{2}[{{{\mbox{V}}}}^{2}]$$

The energy is a function of the capacitance (*C*) and voltage (*V*) of the capacitor. However, in the case of a supercapacitor, the voltage is defined by the electrochemical stability at the electrolyte-electrode interface, and the capacitance is described by another eq.:

2 $$C=\frac{{\varepsilon }_{0}\cdot {\varepsilon }_{r}A}{d}$$

Capacitance (*C*) can thus be increased by either enlarging the electrodes’ surface area (*A*) or decreasing the distance between the electrolyte’s ions and the electrode’s walls (*d*), as the absolute permittivity of the dielectric material being used (*ε*_*r*_) and the vacuum permittivity (*ε*_*0*_) are constants^2^.

Thus, one would quickly conclude that three main pathways can be considered to increase the energy density of supercapacitors:

1. Increasing the operating voltage of the device,
2. Increasing electrode surface area,
3. Optimizing ion-to-electrode distance through tailoring of material properties.

Considering these aspects, one can find that these approaches are highly important when increasing supercapacitors’ energy density. Indeed, some of the most common ways to increase the energy content include the development of non-aqueous electrolyte solutions with operating voltages up to 4 V^3^, carbon materials with gravimetric capacitances up to 100% higher than the market standard^4^, and carbon-electrolyte combinations with over 50% higher areal capacitances than state-of-the-art materials, achieved through pore size optimization^5^.

It can thus be stated that both academic and industrial researchers have produced numerous patents and publications, discovering multiple well-designed approaches for increasing the energy density of supercapacitors, which in 2010 reached values of 85 Wh/kg on the electrode level or ca. 20 Wh/kg on the industrial device level^6^.

Nevertheless, the many impressive results presented in both publications and patents are not observed in industrial devices, even in the worst-case scenario, i.e., considering a delay for product development. Industrial supercapacitor devices have not changed their material composition since the early 2000s and only incrementally increased their energy density in small steps, going from 7 Wh/L in 2002 (Maxwell Technologies Inc.) to 10 Wh/L in 2016 (Nesscap Energy Inc.). After this period, the energy density in commercially available devices remained relatively stagnant except for one manufacturer announcing in 2021 a 16 Wh/L supercapacitor device (72% increase over former products) using a novel material commercially called “Curved Graphene” that was selected based on results from both academic and industrial research^7–10^.

This brief analysis of state-of-the-art supercapacitor technology suggests that the outcomes of both industrial and academic research activities in the supercapacitor field are not easily translated into innovation. Therefore, we should ask the question - what is the reason for the discrepancy between the promising results obtained during the research stage and the lack of significant performance improvement at the industrial device level?

## Finding out which metrics count

One of the core reasons for the described divergence is the information gap between industry and academia, at least in the field of supercapacitors.

The first part of the information gap relates to how much effort is required to introduce improvements in supercapacitor technology. For example, while a 50% increase in energy density is certainly an impressive improvement, it will only be relevant if a device’s energy enhancement is done within a cost range that is economically sustainable for a customer. In more general terms, research is more likely to turn into an innovation only if a novelty can commercially offset the efforts required to obtain it.

**Fig. 2:**
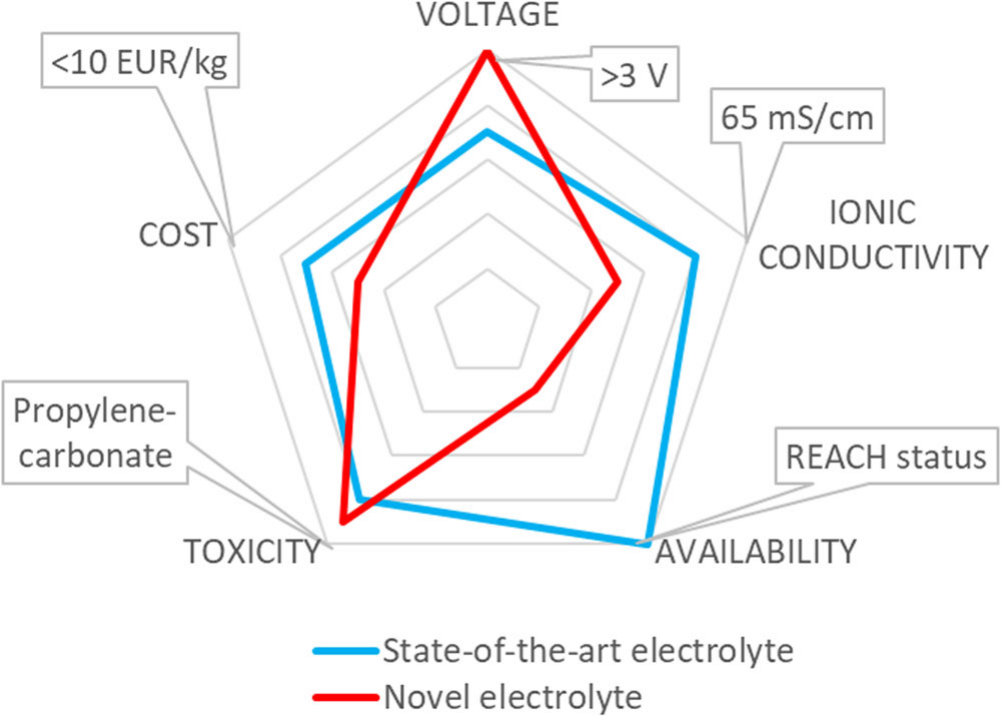
Comparison between a fictional novel electrolyte and a state-of-the-art electrolyte. The innovation potential increases going from the inner to the outer regions of the radar chart. Target values are chosen based on best available option on the market: 3.0 V maximum operating voltage (maximum cell voltage for industrial supercapacitor devices), 65 mS/cm ionic conductivity (using Acetonitrile as a solvent in electrolytes), registered REACH (Registration, Evaluation, Authorisation and Restriction of Chemicals) status for commercial availability, toxicity comparable to Propylene Carbonate (electrolyte component used in various commercial electrochemical energy storage devices) and <10 EUR/kg material cost (cost target for industrial electrolytes).

This means that to turn research into innovation, not only cost but process requirements, general applicability in industrial development, and suitability for end-customer applications must be considered. As an example, Fig. 2 shows a comparison between a state-of-the-art electrolyte and a fictional novel electrolyte. Although the novel electrolyte provides a high operating voltage, its availability is not cost-effective, and the cost and conductivity aspects are not convenient compared to the state-of-the-art equivalent. Thus the innovation potential of the fictional novel electrolyte is severely reduced. Furthermore, while a high operating voltage seems like a good deal at first glance, the inadequate electrolyte ionic conductivity (and therefore the power density) can easily result in a drawback for all applications reliant on power density over energy density.

Another example of an application-based metric for evaluation is the introduction of novel conductive salts in the electrolyte formulation. In particular, while a conductive salt has to be procured and purchased per kg, its function in the device is provided per mole. Thus, a high molar weight can lead to higher production costs, even at a lower cost per weight.

This brief example clearly highlights that, when metrics are considered, it is essential to carefully evaluate the cost of each improvement, whether monetary or technical, against application requirements.

## Material innovation is more than active materials

In the previous example, a typical bottom-up approach has been discussed to increase the energy density of a supercapacitor. However, a top-down approach is often also considered to improve already industrialized devices. In this case, the existing device is analyzed for any areas which could still be improved. This latter approach relates to the second part of the abovementioned information gap.

**Fig. 3:**
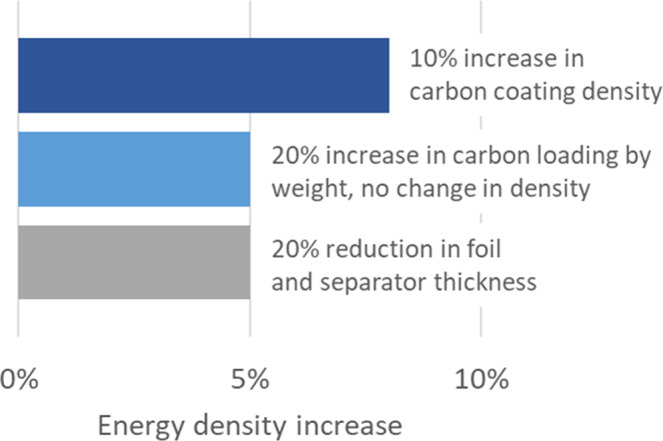
Examples of possible energy density increase strategies for industrial supercapacitors. The data are calculated separately (i.e., the cumulative effect is not considered), taking into account inactive materials and manufacturing processes changes. The calculations are carried out using an industrial cylindrical wound core cell model developed by Skeleton Technologies GmbH. The model specifications are property of Skeleton Technologies GmbH and not publicly available.

In the case of supercapacitors, the top-down approach requires an analysis of the industrial device design to identify the possibility for energy density improvement. Industrial supercapacitors are generally assembled through the winding of positive and negative electrodes, separated by a cellulose separator, resulting in an electrode assembly with a cylindrical shape. The electrodes comprise aluminum foil (i.e., the current collector) coated with microporous carbons (i.e., the charge storage active material)^1^. One way of increasing the energy density of such a device with minimal effort is to decrease the thickness of both the separator and the current collector, or increase the thickness and density of the carbon coating. Both approaches lead to an increased amount of carbon material in the device, thus increasing the stored energy. Fig. 3 shows an example set of results of energy density increase in case of (i) a 20% reduction in Al foil and separator thickness, (ii) a 20% increase in carbon loading of the electrode by weight per area without a change in density and, (iii) a 10% increase in carbon coating density (as weight per volume).

The obvious choice for capacitance improvement for industrial supercapacitor producers has been, for a long time, the optimization of cell and electrode design, especially for non-electrochemical active materials, instead of considering a change of active materials. Indeed, the latter requires extensive testing and material qualification, including a complete overhaul of the supply chain, while the former can generally be done with relatively little effort, sometimes by simply changing a manufacturing machine’s setting.

A similar view can be taken on increased operating voltages. For example, between 2002 and 2016 (see Supplementary Note 1 in the Supplementary Information for details), maximum operating voltages were considered key contributors to the energy density increase in industrial supercapacitors as they rose from 2.5 V (Maxwell, 2002) to 3.0 V (Nesscap, 2016). However, neither electrolyte nor carbon materials had to be completely exchanged to achieve these values. While the material mixtures remain proprietary to the affiliated companies, the most prominent 3.0 V industrial supercapacitors on the market (e.g., LS-Mtron^11^, Skeleton Technologies^7^) still utilize acetonitrile and ammonium salts in the electrolyte formulation. This increase in operating voltage can be partially attributed to high levels of purity in the electrode and cell assembly as well as the electrolyte obtained through improved manufacturing conditions and supply chain quality. Another major contribution to the increase in operating voltage was the introduction of asymmetric electrode capacitances so that the positive and negative electrode’s potential range in an electrolyte mixture could be fully exploited, avoiding degradation. This innovation was successfully carried over from earlier research works, where an essential contribution came from a better understanding of degradation mechanisms in industrial supercapacitors^12^. Since then, the asymmetric setup of carbon-carbon supercapacitor devices has become a standard in the industry and was also employed to increase the maximum operating voltage on the device level^13,14^.

## Utilizing methods that are as close to the application as possible

While the supercapacitor voltage in industrial devices has been increased from 2.5 to 3.0 V, results reported in academic literature seem to be vastly superior, with values of over 4.0 V being reported. The reason for this discrepancy can be found in the difference between the characterization methods used in academia and industry.

As industrial supercapacitors are commonly used in applications which require a constant voltage to be kept over time, the defining characterization method for evaluation of maximum operating voltage follows a constant voltage approach, in which aging is accelerated by using high temperatures^15^.

However, research literature often utilizes constant galvanostatic cycling or cyclic voltammetry to evaluate maximum operating voltage. From an industrial perspective, these methods provide little insight into the stability of the electrolyte-electrode interface in the application, even less if the electrodes consist of glassy carbon or metals and are not directly comparable to electrodes employed in industrially-produced devices. Thus, constant voltage experiments must be part of any study which claims improved operating voltage and device lifetime^15,16^.

Similar issues can be found when evaluating capacitance where the volumetric capacitance is the most relevant characteristic, as the volume in an industrially-produced device is limited. Furthermore, to allow for a direct comparison to performance in application, both gravimetric and volumetric capacitance should relate to the weight and volume of all electrode materials (i.e., binder, conductive additives, and active material).

Thus, the methods and experimental setups employed have a significant impact on the transition of research into innovation.

## Innovation through scientific peer-reviewed publication requires proper data reports and analyses

Another significant difference between research in academia and industry is found in the measurement of success. “Publish or perish” philosophy is crucial in the academic world, and success is measured in the quality and quantity of papers published and patents filed. In industrial research, however, the key performance indicators are centered around the resulting innovation, meaning that the experimental results must stand the test of practical application.

One issue with some publications in the field of energy storage is that research results are presented as highly innovative, mainly to show that above-average performance is achieved or that a breakthrough innovation is around the corner. This issue is well-known in academia, as discussed in a recent editorial by Johansson et al.^17^, and it is clear that unbiased data reports, analyses, and discussions are required to prevent this issue from harming practical innovation efforts.

As measurement techniques and experimental setups can heavily influence the results achieved, it is possible to show excellent performance by choosing one measurement technique over the other.

A relevant example in the electrochemical energy storage field is the use of thin-coated electrodes (<20 µm) to improve power and energy density. While such approaches certainly yield valuable data, the individual performance results must be discussed critically, and impacts on industrialization should be mentioned. In cases where these aspects are not openly addressed, the corresponding results could lead other researchers (either academic or industrial) to divert their scientific efforts towards paths they otherwise would not have pursued. Also, in some cases, academic researchers focus on future publications aiming to outperform state-of-the-art research articles. This aspect is also risky, as hastily carried out studies can easily create a “technology debt” that steadily increases with each publication trying to outperform the previous one.

Analysis of data collected from measurements carried out in relevant practical conditions is more helpful to create innovation than data obtained with the aim to look unprecedented or outperforming. Even if showing that an idea does not work or that a material is worse than the state-of-the-art, there is still a significant scientific value in it, especially for designers of new applications.

## Summary and outlook

Closer cooperation between industry and academia, also in the form of joint projects funded by industrial partners, could accelerate the creation of innovation from research, considering that data produced from such types of projects are reviewed from an application-based standpoint.

However, even in the ideal case of “perfect” cooperation between the various project partners, the transition from research to innovation can only happen if meaningful data are collected and analyzed. This meaningful data must be obtained using application-based measurements and adequate design of experiments. With the supercapacitor research example discussed here, the author of this article hopes to give some insight into the importance of an application-based approach. In order to support innovation with research, application-based characterization and measurement approaches, meaningful statistical data, and publication of “negative” results are thus invaluable tools and should be disclosed and discussed as often as possible within and between the various research communities.

## Supplementary information

Supplementary Information

## References

[1] Schütter C, Pohlmann S, Balducci A (2019). Industrial requirements of materials for electrical double layer capacitors: impact on current and future applications. Adv. Energy Mater..

[2] Conway, B. *Electrochemical supercapacitors*. (Kluwer Acad., 2009).

[3] Li J, Wang N, Tian J, Qian W, Chu W (2018). Supercapacitors: cross-coupled macro-mesoporous carbon network toward record high energy-power density supercapacitor at 4 V. Adv. Funct. Mater..

[4] Xu B (2008). Highly mesoporous and high surface area carbon: A high capacitance electrode material for EDLCs with various electrolytes. Electrochem. Commun..

[5] Chmiola J (2006). Anomalous increase in carbon capacitance at pore sizes less than 1 nanometer. Science.

[6] Liu C, Yu Z, Neff D, Zhamu A, Jang BZ (2010). Graphene-based supercapacitor with an ultrahigh energy density. Nano Lett..

[7] SkelCap SCX Series Ultracapacitor Cells. *Skeletontech.com* (2021). at https://www.skeletontech.com/skelcap-scx-ultracapacitor-cells, accessed on 28.01.2022.

[8] Arulepp M (2006). The advanced carbide-derived carbon based supercapacitor. J. Power Sources.

[9] Madiberk, V., Leis, J., Arulepp, M., Rumma, K. & Perkson, A. Super capacitor of high specific capacity and energy density and the structure of said super capacitor. Patent US9111693B2 (2010). https://patents.google.com/patent/US9111693B2/en, accessed on 28.01.2022.

[10] Leis, J., Arulepp, M. & Perkson, A. Electrical double layer capacitor with enhanced working voltage. Patent US8911510B2 (2011). https://patents.google.com/patent/US8911510B2/en, accessed on 28.01.2022.

[11] Ultracapacitor products, lsmtron.com/ (2022). at https://www.lsmtron.com/page/popup/productAllView.asp?divisionCode=D0700, accessed on 28.01.2022.

[12] Kötz R, Ruch P, Cericola D (2010). Aging and failure mode of electrochemical double layer capacitors during accelerated constant load tests. J. Power Sources.

[13] Lazzari M, Soavi F, Mastragostino M (2008). High voltage, asymmetric EDLCs based on xerogel carbon and hydrophobic IL electrolytes. J. Power Sources.

[14] Pohlmann S, Balducci A (2013). A new conducting salt for high voltage propylene carbonate-based electrochemical double layer capacitors. Electrochim. Acta.

[15] Ruschhaupt P, Pohlmann S, Varzi A, Passerini S (2020). Determining realistic electrochemical stability windows of electrolytes for electrical double‐layer capacitors. Batteries Supercaps.

[16] Weingarth D, Noh H, Foelske-Schmitz A, Wokaun A, Kötz R (2013). A reliable determination method of stability limits for electrochemical double layer capacitors. Electrochim. Acta.

[17] Johansson P (2021). Ten ways to fool the masses when presenting battery research. Batteries Supercaps.

