# Supplementary material for: Metrics and methods for moving from research to innovation in energy storage
Source: Nat Commun. 2022 Mar 22;13:1538. doi: 10.1038/s41467-022-29257-w (PMC8941066; doi:10.1038/s41467-022-29257-w)
Supplement: Supplementary file 1 — Supplementary Information [file 41467_2022_29257_MOESM1_ESM.pdf]

Supplementary Information

Metrics and methods for moving from research to innovation in energy storage

Sebastian Pohlmann[a]

[a] Skeleton Technologies OÜ, Valukoja 8, 11415 Tallinn, ESTONIA sebastian.pohlmann@skeletontech.com

Supplementary Note 1

The evolution of historical specifications for industrial supercapacitors between 2002 and 2016 discussed under the paragraph “Material innovation is more than active materials” was determined using the “internet archive” (https://web.archive.org/) and drawn from historical records of both Maxwell Technologies Inc. and Nesscap Energy Inc. It can be seen that most advances in energy density were based on voltage increase.

All website records were last accessed by the author on 28.01.2022.

Energy density for Maxwell Technologies Inc. products 2002 (2500 F, 2.5 V, 725 g, 609 mL, 3.5 Wh/L):

https://web.archive.org/web/20021207090545/http://www.maxwell.com/ultracapacitors/products/large_cell.html

Energy density for Maxwell Technologies Inc. products 2005 (2600 F, 2.7 V, 470 g, 6.2 Wh/L):

https://web.archive.org/web/20051228145108/http://www.maxwell.com/ultracapacitors/products/large_cell.html

Energy density for Nesscap Energy Inc. products 2016 (3400 F, 3.0 V, 390 mL, 10.7 Wh/L):

https://web.archive.org/web/20161129201846/http://www.nesscap.com/

Operative voltage for Maxwell Technologies Inc. products 2002 (2.5 V):

https://web.archive.org/web/20021207090545/http://www.maxwell.com/ultracapacitors/products/large_cell.html

Operative voltage for Nesscap Energy Inc. products 2016 (3.0 V):
https://web.archive.org/web/20161129201846/http://www.nesscap.com/
